# In the field of membrane protein structural biology, chance only favors the sample which is properly prepared

**DOI:** 10.1063/4.0000820

**Published:** 2025-10-27

**Authors:** Vikas Navratna

**Affiliations:** University of Michigan, Ann Arbor, MI, USA

## Abstract

Small membrane proteins (<100 kDa) are most often ensconced entirely within the plane of the lipid bilayer and are sensitive to changes in their native environment, making them unstable and recalcitrant targets of X-ray crystallography upon being extracted from the membrane. In the past decade, single particle cryo-electron microscopy (cryo-EM) has established itself as the method of choice for determination of high-resolution small membrane protein structures, primarily because it negates the requirement of high-quality diffracting crystals. However, the lack of conspicuous extra-membranous soluble domains, and the lack of asymmetry in the shape of the small membrane proteins by virtue of them being embedded entirely within detergent micelles, can affect particle alignment and cause challenges in data processing. Overcoming these challenges may require a combination of strategies that include protein expression construct engineering, optimization of sample preparation conditions, reconstitution of proteins in membrane mimetic environments, complexation with small molecule ligands and fiducial markers that can enhance the protein stability and size, and even employment of electron detectors with optimal detective quantum efficiency performance. My talk will use examples from my firsthand experience to demonstrate the applicability of these strategies to discover novel aspects, albeit sometimes serendipitously, of different classes of small membrane proteins from humans.

